# *PHF6* Degrees of Separation: The Multifaceted Roles of a Chromatin Adaptor Protein

**DOI:** 10.3390/genes6020325

**Published:** 2015-06-19

**Authors:** Matthew A.M. Todd, Danton Ivanochko, David J. Picketts

**Affiliations:** 1 Regenerative Medicine Program, Ottawa Hospital Research Institute, Ottawa, Ontario, K1H 8L6, Canada; E-Mails: mtodd.research@gmail.com (M.A.M.T.); divan061@uottawa.ca (D.I.); 2 Department of Biochemistry, Microbiology, and Immunology, Faculty of Medicine, University of Ottawa, Ontario, K1H 8M5, Canada; 3 Department of Cellular and Molecular Medicine, Faculty of Medicine, University of Ottawa, Ontario, K1H 8M5, Canada

**Keywords:** AML, BFLS, hematopoiesis, neurogenesis, nucleolus, NuRD, PAF1, PHF6, T-ALL, XLID

## Abstract

The importance of chromatin regulation to human disease is highlighted by the growing number of mutations identified in genes encoding chromatin remodeling proteins. While such mutations were first identified in severe developmental disorders, or in specific cancers, several genes have been implicated in both, including the *plant homeodomain finger protein 6* (*PHF6*) gene. Indeed, germline mutations in *PHF6* are the cause of the Börjeson–Forssman–Lehmann X-linked intellectual disability syndrome (BFLS), while somatic PHF6 mutations have been identified in T-cell acute lymphoblastic leukemia (T-ALL) and acute myeloid leukemia (AML). Studies from different groups over the last few years have made a significant impact towards a functional understanding of PHF6 protein function. In this review, we summarize the current knowledge of PHF6 with particular emphasis on how it interfaces with a distinct set of interacting partners and its functional roles in the nucleoplasm and nucleolus. Overall, PHF6 is emerging as a key chromatin adaptor protein critical to the regulation of neurogenesis and hematopoiesis.

## 1. Introduction

Mutations in proteins that chemically modify chromatin, or possess reader domains to bind these modifications are now documented in a growing number of developmental diseases [1]. More recently, the high-throughput screening of various cancer genomes using next-generation screening technologies (e.g., Roche/454, Illumina/Solexa) has yielded mutations in many of these same epigenetic regulators [2,3,4]. These mutations target genes encoding proteins that are responsible for ATP-dependent nucleosome reorganization (e.g., ATRX), histone tail modifiers (e.g., JARID1C), histone variants (e.g., histone H3.3), DNA methyltransferases (e.g., DNMT3A/B), and proteins that interface with chromatin (e.g., MECP2) [4,5,6,7,8]. Here, we review recent advances in a gene that encodes another such protein, *plant homeodomain finger protein 6* (*PHF6*), which possesses two chromatin-binding zinc finger domains and has been implicated in the Börjeson–Forssman–Lehmann X-linked intellectual disability syndrome (BFLS), T-cell acute lymphoblastic leukemia (T-ALL), and acute myeloid leukemia (AML) [9,10,11].

### 1.1. Structure and Expression Pattern of PHF6

**Figure 1:**
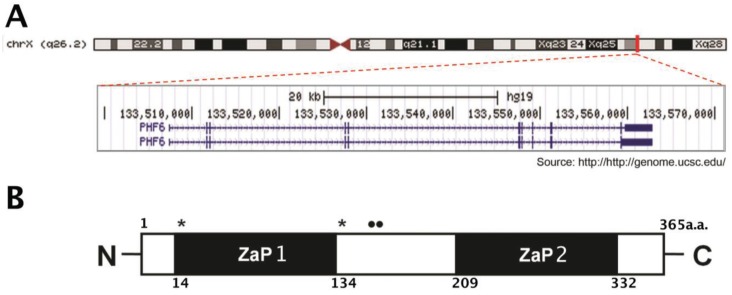
*PHF6* gene and protein domain structures. (**A**) The *PHF6* gene contains 11 exons and is located on the X chromosome. Source: UCSC Genome Browser [21]. (**B**) The gene encodes a protein of 365 amino acids with two ZaP (zinc knuckle, atypical PHD) domains and localization signals for the nucleus (_*_) and nucleolus (••).

The *PHF6* (Gene ID: 84295) gene is located on the X chromosome, consists of 11 exons, and is transcribed into a 4.5 kb mRNA (see Figure 1A). Exons 2–10 encode a 365 amino acid (41 kDa) protein (Uniprot: Q8IWS0) (see Figure 1B), while exons 1 and 11 comprise the 5’ and 3’ untranslated regions (UTRs), respectively. Two mRNA isoforms exist in humans, with the second incorporating intron 10 to increase the size of the 3’ UTR [9]. A third isoform is predicted to encode a truncated 312 amino acid version of the PHF6 protein, but its existence has not been confirmed experimentally [12]. The PHF6 protein is highly conserved among vertebrates, with 97.5% amino acid identity between humans and mice. Invertebrates do not have a *PHF6* homolog. Structurally, the most prominent features of PHF6 are its two nearly identical zinc finger domains (ZaP1: aa 14–134; ZaP2: aa 209–332) that are derived from a PZP motif [13]. PZP motifs consist of a PHD domain, followed by a zinc knuckle, followed by an atypical PHD domain, however each zinc finger domain of PHF6 is a degenerate version of this structure as they consist only of the zinc knuckle and the atypical PHD (ZaP) [14]. PHF6 also contains two nuclear localization sequences (NLS1: aa 13–16; NLS2: aa 129–133) and a nucleolar localization sequence (NoLS: 157–169), with the localization of PHF6 to the nucleus and nucleolus having been confirmed by immunocytochemistry, subcellular fractionation, and mass spectrometry-based identifications [9,14,15,16,17,18].

To date, no *Phf6* animal knockout models have been published and few studies record its gene expression patterns during embryonic and postnatal mouse development. In mice, *Phf6* expression has been observed to be ubiquitous, with particularly high expression in the brain and central nervous system (CNS) throughout embryonic development, and more moderate expression during post-natal development and in adult tissues [19]. Particularly high PHF6 expression is observed in the cortical plate and intermediate zone at E14.5, as well as the ventricular and subventricular zones by E16.5 [20]. *Phf6* is also highly expressed in other embryonic tissues, including the anterior pituitary at E12.5, nasal processes from E9.5 to E12.5, pharyngeal arches at E9.5, and limb buds at E12.5. In another study, *Phf6* expression was elevated in murine T-cell lymphoma, supporting observations of high *PHF6* expression in human adult B- and T-lymphoid cells [11,15]. Thus, collectively, these expression studies are indicative of a potential role for PHF6 during both neurogenesis and hematopoiesis.

### 1.2. Regulation of PHF6 Transcripts

The epigenetic mechanisms that regulate *PHF6* transcription have not been well characterized aside from the identification of a NOTCH1 binding site and DNA methylation sites in the *PHF6* promoter [22,23]. Post-transcriptionally, *PHF6* mRNA is targeted by as many as 25 microRNAs, including miR-20a, miR-26a, miR-128, and miR-574 [20,24,25]. Franzoni *et al.* recently described that miR-128, which has three binding sites in the *Phf6* 3’ UTR, is essential for mediating the switch between neuronal migration and neurite outgrowth by silencing *Phf6* in upper level cortical neurons [20]. Indeed the downregulation of *Phf6* that occurs in these cortical layers during development was inversely correlated with an increase in the expression levels of miR-128. Interestingly, miR-128 is also oncogenically expressed in tumours arising from non-neuronal tissue, including T-ALL, where *PHF6* was confirmed as a target [25].

## 2. Consequences of Germline Mutations of *PHF6*

### 2.1. Börjeson–Forssman–Lehmann Syndrome (BFLS)

The BFLS phenotype was first reported a half century ago and the multiple variable aspects of the phenotype have been well described in the literature [26,27,28,29,30]. The majority of BFLS cases occur in males who display developmental delay within their first year after birth, with mild to severe intellectual disability becoming apparent during childhood and adolescence [27,28]. Additional features of the BFLS phenotype include large ears, coarse facial features (e.g., deep set eyes), long tapered fingers, syndactyly and shortening of the toes, gynecomastia, truncal obesity, hypogonadism, hypotonia, and feeding problems during infancy. Some features are present at birth (e.g., hypogonadism), however other aspects of the phenotype present themselves during childhood and adolescence (e.g., coarse facial features, truncal obesity). Gynecomastia in particular emerges during adolescence, although it is unclear if it is caused by breast tissue hyperplasia or lipomastia [29]. Less common features of BFLS include microcephaly/macrocephaly, short stature, epilepsy, cleft lip and palate, hearing impairment, and hypopituitarism [28]. Two independent probands have also been diagnosed with bone marrow cancers, including Hodgkin’s lymphoma and T-ALL [29,31]. Female carriers within BFLS families typically display mild, if any, symptoms, with at least one isolated female patient expressing a *de novo PHF6* mutation having been diagnosed with BFLS marked by mild intellectual disability [32].

While BFLS was long known to be X-linked, having been previously mapped to the Xq26–27 chromosomal region, it was not until 2002 that *PHF6* mutations were identified as its cause [9,33,34,35]. These mutations include missense mutations, nonsense mutations, and deletions (see Table 1). These mutations are distributed across the entire *PHF6* gene, suggesting that the associated BFLS phenotypes arise from a loss of PHF6 function, however attempts to identify genotype-phenotype correlations between specific *PHF6* mutations and the severity of intellectual disability have not been successful [29,30]. To date, no other gene mutations are known to cause BFLS, however not all BFLS patients have an identified *PHF6* mutation. In one cohort, for instance, *PHF6* mutations were only identified in 5/25 patients, indicating that there could be additional BFLS loci, or more likely, that the clinical diagnosis of BFLS overlaps with other syndromes [32]. In this regard, many X-linked intellectual disability syndromes (e.g., Coffin–Lowry syndrome, Klinefelter syndrome, Prader–Willi syndrome, Wilson–Turner syndrome) share overlapping phenotypes with BFLS such as gynecomastia, obesity, hypotonia, hypogonadism, or digit abnormalities [28,36].

### 2.2. Coffin–Siris syndrome

First described in 1970, Coffin–Siris syndrome (reviewed by [37,38]) is an intellectual disability disorder that is caused by mutations in genes encoding individual subunits of the BAF (BRG1/BRM-associated factor) complex, including *ARID1A*, *ARID1B*, *SMARCA2*, *SMARCA4* (*BRG1*), *SMARCB1* (*INI1*), and *SMARCE1* (*BAF57*) [39,40]. Interestingly, several recent studies describe female patients expressing *de novo PHF6* mutations that exhibit more severe intellectual disability than female BFLS carriers, overlapping more closely with Coffin–Siris syndrome than with features of classical BFLS [30,41,42,43,44]. Distinguishing features of this newly described phenotype include sparse hair, deep set eyes, hypoplasia of the fifth digit, linear skin hyperpigmentation, and dental abnormalities [44]. Unlike male BFLS patients, these females do not display truncal obesity and syndactyly occurs between the third and fifth toes, rather than between the second and third toes [42,44]. Notably, *de novo PHF6* mutations in females primarily consist of deletions or frameshift mutations (see Table 1) in comparison to male BFLS patients or female carriers, which more commonly express point mutations. Furthermore, it is tantalizing to speculate that these more deleterious mutations might be lethal in males.

Female patients with *de novo PHF6* mutations are nearly indistinguishable from Coffin–Siris syndrome patients during early infancy, but develop a more distinct phenotype as they age, such as the display of linear skin hyperpigmentation, which does not occur in Coffin–Siris syndrome patients [30,41]. Interestingly, females that lack linear skin hyperpigmentation exhibit a phenotype that more closely resembles classical BFLS than the Coffin–Siris-like phenotype. Indeed, Zweier *et al.* argue that functional mosaicism from variable rates of skewed X-chromosome inactivation in different tissues drives the Coffin–Siris-like phenotype [30]. In this regard, every documented case of a female patient expressing a *de novo PHF6* mutation has presented with skewed X-chromosome inactivation (in peripheral blood) of 93% or higher, yet they present with a severe phenotype [30]. However, analysis of fibroblast cells in some Coffin–Siris-like patients has demonstrated variable X-inactivation (58%–83%) suggesting that reduced X-inactivation skewing in other tissues is what contributes to the phenotypic differences [42]. Consistent with this idea, there is no correlation with the level of skewing in peripheral blood cells in BFLS carriers with or without a phenotype [29,42,43]. Therefore, whether or not female patients expressing a *PHF6* mutation develop BFLS or the Coffin–Siris-like phenotype may depend upon the type of mutation (deletion *vs.* point mutation) and on the extent to which skewed X-chromosome inactivation occurs in different tissues.

**Table 1:** Summary of germline *PHF6* mutations.

Gender	Nucleotide Change	Amino Acid Change	Type of Mutation	Location of Mutation	Cancer	Isolated^3^/ *De novo*	Reference
M	c.2T>C	p.M1T	Missense	Exon 2			[9]
M	c.2T>C	p.M1T	Missense	Exon 2			[32]
M	c.134G>A	p.C45Y	Missense	Exon 2			[9]
M	c.134G>A	p.C45Y	Missense	Exon 2		Isolated	[9]
M	c.266G>T	p.G89V	Missense	Exon 4			[45]
M	c.296G>T	p.C99F	Missense	Exon 4		Isolated	[9]
M, F	c.686A>G	p.H229R	Missense	Exon 7			[9]
M	c.700A>G	p.K234E	Missense	Exon 7			[9]
M	c.769A>G	p.R257G	Missense	Exon 8			[9]
M	c.769A>G	p.R257G	Missense	Exon 8	Yes^2^	Isolated	[16]
M	c.940A>G	p.I314V	Missense	Exon 9			[32]
M	c.22A>T	p.K8*	Nonsense	Exon 2			[9]
F	c.955C>T	p.R319*	Nonsense	Exon 9		De novo	[42]
M	c.1024C>T	p.R342*	Nonsense	Exon 10		De novo	[26,28]
M	c.1024C>T	p.R342*	Nonsense	Exon 10			[9]
M	c.1024C>T	p.R342*	Nonsense	Exon 10			[9]
M	c.1024C>T	p.R342*	Nonsense	Exon 10			[9]
M	c.1024C>T	p.R342*	Nonsense	Exon 10	Yes^3^		[31]
F	c.27dupA	p.G10fs*21	Frameshift	Exon 2		De novo	[32]
M	IVS2–8A>G	M46fsΔexon3	Frameshift	Exon 3			[16]
F	c.677delG	p.G226fsE*53	Frameshift	Exon 7		De novo	[41]
F	c.914G>T	p.C305F	Frameshift	Exon 9		De novo	[41]
F			Duplication	Exons 4–5		De novo	[42]
F			Duplication	Exons 4–5		De novo	[42]
F	6 kb deletion		Deletion	Exons 4–5		De novo	[42]
F	100 kb deletion		Deletion	Exons 6–10		De novo	[44]
F	15 kb deletion		Deletion	Exons 9–11		De novo	[43]
M	c.999–1001 delTGA	p.D333del	Deletion	Exon 10			[46]
M	c.999–1001 delTGA	p.D333del	Deletion	Exon 10			[26,28]
F	Entire gene deleted		Deletion	Whole gene		De novo	[42]
F	270 kb deletion		Deletion	Whole gene		Isolated	[44]

^1^ Isolated refers to instances where the parents have not been screened for *PHF6* mutations. ^2^ Hodgkin’s lymphoma ^3^ T-cell acute lymphoblastic leukemia

## 3. Consequences of Somatic Mutations of *PHF6*

### 3.1. T-Cell Acute Lymphoblastic Leukemia (T-ALL)

Somatic *PHF6* mutations in human tumours were first described for T-ALL patients [10,31]. Van Vlierberghe and colleagues reported *PHF6* mutations in 16% of pediatric and 38% of adult subjects. In four additional studies, other groups have identified T-ALL-related *PHF6* mutation frequencies varying from 5% to 40% within each cohort [47,48,49,50]. While Van Vlierberghe *et al.* reported a significantly higher incidence of PHF6 mutations among males, gender differences were not observed in these later studies. In contrast to male BFLS patient mutations, which are primarily missense, neoplastic *PHF6* mutations overwhelmingly consist of deletions, frameshifts, nonsense mutations, or missense mutations that target zinc ion stabilizing residues in the second ZaP domain (see Table 2). Collectively, these findings suggest that PHF6 is a tumour suppressor and that these mutations result in a loss of function, consistent with silencing of the *PHF6* promoter by DNA methylation in some T-ALL tumours [23].

T-ALL arises from developmentally arrested immature T-cells that express one or more mutations to bypass key developmental checkpoints (*i.e.* β selection, -/+ selection) during thymocyte maturation [51]. Many of the mutations contributing to T-ALL involve oncogenic translocation events at the T-cell receptor (TCR) gene loci and are categorized within two groups: (i) those that initiate oncogenesis and define the molecular-genetic subtype of the tumour, and (ii) mutations that are recurrent [52]. Major T-ALL subtypes are defined by aberrant oncogenic activation or chromosomal translocation events in genes coding for bHLH proteins (e.g., TAL1), LMO proteins (e.g., LMO1), homeobox proteins (e.g., TLX1, TLX3), or proto-oncogenes (e.g., c-MYB), while recurrent mutations target cell signalling pathways (e.g., NOTCH1, signal transduction), cell cycle regulatory mechanisms, and tumour suppressors (*i.e*. for inactivation) [52]. Interestingly, *PHF6* mutations are associated with tumours expressing the *TLX1* and *TLX3* oncogenes [10]. Consistent with this observation, *PHF6* is highly expressed in DP cells, the stage at which *TLX1*-induced tumours most commonly experience developmental arrest [10,53]. Moreover, *PHF6* has also been demonstrated to be a direct target of *TLX1*-mediated repression [54]. *PHF6* mutations are also co-expressed with JAK1 mutations, SET-NUP214 translocations, and activating *NOTCH1* mutations, which occur in over half of all T-ALL patients [48,50,55].

### 3.2. Acute Myeloid Leukemia (AML)

*PHF6* mutations have also been identified in AML patients (see Table 2) [11]. AML is a heterogeneous cancer that develops in multiple progenitor cell types of the myeloid lineage. Recent profiling of the AML cancer genome with next-generation sequencing has demonstrated that most AML mutations are recurrent, consisting primarily of chromosomal translocation events at specific loci and targeted mutations among a group of 23 genes, including epigenetic regulators such as DNMT3A, TET2, and RUNX1 [8]. Van Vlierberghe *et al.* found *PHF6* mutations in 10/353 patients. Similar to T-ALL, *PHF6* mutations primarily consisted of deletions, frameshifts, nonsense mutations and missense mutations targeting zinc ion-stabilizing residues from the second ZaP domain. Additional AML screens by other groups found *PHF6* mutations in 3% of screened tumours [8]. Additionally, *PHF6* mutations correlated with reduced overall survival in adult patients and were observed to be co-expressed with *RUNX1* mutations [8,56]. As of yet, tumours expressing *PHF6* mutations have not been correlated with any specific myeloid progenitor cell type, however *PHF6* expression levels were observed to be higher in hematopoietic stem cells and megakaryocyte/erythroid progenitors than in common myeloid progenitors or granulocyte/macrophage progenitors [11].

### 3.3. PHF6 Loss-of-Function in Other Neoplasias

Isolated cases of loss-of-function *PHF6* mutations in chronic myeloid leukemia (CML) and hepatocellular carcinoma patient tumours have also been reported [49,57]. Interestingly, shRNA-mediated knockdown of *Phf6* in a murine B-cell acute lymphoblastic leukemia (B-ALL) model was observed to significantly reduce the rates of tumour proliferation, in contrast to both T-ALL and AML models, where the loss of *Phf6* correlates with enhanced tumour progression [24,58]. Consistent with the B-ALL model, *PHF6* knockdown in a HeLa cell model led to a reduction in rates of cellular proliferation, accompanied by a cell cycle delay at G2/M [59]. Thus, the downstream functions of PHF6 leading to tumour suppression in specific cell or tissue types (e.g., lymphoid and myeloid) are not necessarily limited to proliferative or cell cycle regulatory processes. Rather, PHF6 likely participates in additional development regulatory networks that are context-dependent and tissue-specific, with the tumorigenic outcome being influenced by the presence of additional oncogenic mutations. This pathogenic model is analogous to the example provided by BCL11B in developing thymocytes, in which the loss of BCL11B on its own induces massive apoptosis during β-selection, yet also favours T-ALL progression when lost in combination with other oncogenically favourable events [54,60].

**Table 2:** Summary of cancer-related *PHF6* mutations.

Gender	Nucleotide Change	Amino Acid Change	Type of Mutation	Location of Mutation	Cancer^1^	Reference
F	c.90_91insCCCG	p.L31PfsX6	Insertion/Deletion	Exon 2	T-ALL	[49]
M		p.G10fs	Frameshift	Exon 2	T-ALL	[10]
M		p.A41fs	Frameshift	Exon 2	T-ALL	[10]
M		p.H44fs	Frameshift	Exon 2	T-ALL	[10]
F	c.76-95del20+insTTGG	p.P26fs	Frameshift	Exon 2	T-ALL	[48]
M		p.Y105fs	Frameshift	Exon 3	T-ALL	[10]
M	c.267_268insTTAGGACC	p.A90LfsX10	Insertion/Deletion	Exon 4	T-ALL	[49]
M		p.G122X	Nonsense	Exon 4	T-ALL	[10]
M		p.R116X	Nonsense	Exon 4	T-ALL	[10]
M		p.T98fs	Frameshift	Exon 4	T-ALL	[10]
F	c.289A>T	p.K97X	Nonsense	Exon 4	T-ALL	[48]
M		p.H135fs	Frameshift	Exon 5	T-ALL	[10]
F		p.F172fs	Frameshift	Exon 6	T-ALL	[10]
M		p.R225X	Nonsense	Exon 6	T-ALL	[10]
M		p.S158fs	Frameshift	Exon 6	T-ALL	[10]
M		p.S191fs	Frameshift	Exon 6	T-ALL	[10]
M	c.525_526delGT	p.S176fs	Frameshift	Exon 6	T-ALL	[48]
M	c.673C > T	p.R225X	Nonsense	Exon 7	T-ALL	[49]
M	c.653_667delGGGAGGAAGAAAATGinsCCCTTTAAAGGGA	p.G218AfsX	Insertion/Deletion	Exon 7	T-ALL	[49]
M		p.K235X	Nonsense	Exon 7	T-ALL	[10]
M		p.C215Y	Missense	Exon 7	T-ALL	[10]
M		p.G263fs	Frameshift	Exon 8	T-ALL	[10]
M	c.735M736dupTT	p.S246FfsX34	Duplication	Exon 8	T-ALL	[49]
M		p.K273X	Nonsense	Exon 8	T-ALL	[10]
M		p.C280Y	Missense	Exon 8	T-ALL	[10]
M		p.R257X	Nonsense	Exon 8	T-ALL	[10]
M	c.820T>C	p.R274X	Nonsense	Exon 8	T-ALL	[10]
M	c.808C>T	p.Q270X	Nonsense	Exon 8	T-ALL	[48]
M	c.823G>A	p.G275R	Missense	Exon 8	T-ALL	[48]
M	c.779insCGGGAGGATCC	p.D262fs	Frameshift	Exon 8	T-ALL	[48]
M		p.S320X	Nonsense	Exon 9	T-ALL	[10]
M	c.903C>A	p.Y301X	Nonsense	Exon 9	T-ALL	[49]
M		p.Y303fs	Frameshift	Exon 9	T-ALL	[10]
M		p.C283R	Missense	Exon 9	T-ALL	[10]
M		p.T300A	Missense	Exon 9	T-ALL	[10]
M		p.A311P	Missense	Exon 9	T-ALL	[10]
M		p.Y303X	Nonsense	Exon 9	T-ALL	[10]
M	c.933_934insT	p.A311X	Nonsense	Exon 9	T-ALL	[48]
M	c.835delA	p.K279fs	Frameshift	Exon 9	T-ALL	[48]
M	c.1024C>T	p.R342*	Nonsense	Exon 10	T-ALL	[31]
M		p.D333fs	Frameshift	Exon 10	T-ALL	[10]
M	c.986A>G	p.H329R	Missense	Exon 10	T-ALL	[10]
M	c.973T>C	p.Y325H	Missense	Exon 10	T-ALL	[48]
M		p.C215Y	Missense	Exon 7	T-ALL	[10]
M		p.C28fs	Frameshift	Exon 2	T-ALL	[10]
F	c.968 + 1G > A	Undetermined	Non-coding	Intron 9	T-ALL	[49]
M	c.968+2T_968+5GdelTAAG	Undetermined	Non-coding	Intron 9	T-ALL	[49]
M	0.55 Mb deleted	Absent	Deletion	n/a	T-ALL	[10]
M	0.23 Mb deleted	Absent	Deletion	n/a	T-ALL	[10]
M	1.50 Mb deleted	Absent	Deletion	n/a	T-ALL	[10]
M	0.27 Mb deleted	Absent	Deletion	n/a	T-ALL	[10]
M	1.90 Mb deleted	Absent	Deletion	n/a	T-ALL	[10]
M	0.20 Mb deleted	Absent	Deletion	n/a	T-ALL	[10]
M	0.08 Mb deleted	Absent	Deletion	n/a	T-ALL	[10]
M	0.11 Mb deleted	Absent	Deletion	n/a	T-ALL	[10]
M		p.C20fs	Frameshift	Exon 2	AML	[11]
M		p.A40G	Missense	Exon 2	AML	[11]
F		p.N171fs	Frameshift	Exon 6	AML	[11]
M		p.P200fs	Frameshift	Exon 7	AML	[11]
M		p.R274X	Nonsense	Exon 8	AML	[11]
M		p.R335fs	Frameshift	Exon 9	AML	[11]
M		p.H302Y	Missense	Exon 9	AML	[11]
M		p.R319X	Nonsense	Exon 9	AML	[11]
M		p.H329L	Missense	Exon 10	AML	[11]
M		p.R342X	Nonsense	Exon 10	AML	[11]
M	c.27dupA	p.K9RfsX12	Duplication	Exon 2	AML	[49]
M	c.83_101delGTGGACAGTTACTAATATCinsAT	P.C28YfsX2	Insertion/Deletion	Exon 2	AML	[49]
M	c.769A>G	p.R257G	Missense	Exon 8	HA	[16]
M	c.673C > T	p.R225X	Nonsense	Exon 7	HC	[9]
M	c.665C>T	p.A135V	Missense	Exon 5	CML	[57]
M	c.670–679del10	p.N137_E139del140fsX142	Frameshift	Exon 5	CML	[57]
M	c.895–896delTG	p.C212WfsX222	Frameshift	Exon 7	CML	[57]

^1^ HA = Hodgkin’s lymphoma; HC = Hepatocellular carcinoma; CML = Chronic myeloid leukemia

## 4. Delineating the Functional Interactions of PHF6

Until relatively recently, the role of PHF6 was categorized based on its homology with chromatin remodelling proteins, however several recent studies have shed an early light upon how its molecular responsibilities may contribute to the developmental processes of neurogenesis and hematopoiesis; activities that become compromised during development and/or in cancer. These studies included structural characterization of the PHF6 zinc finger domain, nuclear and nucleolar localization studies, the identification of PHF6-containing protein complexes, and reports of putative phosphorylation sites.

### 4.1. Functional Analysis of the Conserved Motifs within PHF6

In humans, PZP/ZaP domains are primarily found in proteins that participate in transcriptional regulation or changes to chromatin structure (e.g., histone acetylation, methylation, demethylation) (see Table 3) [14]. Canonical PHD domains are Cys4-His-Cys3 type zinc fingers that chelate two zinc ions and participate in protein–protein interactions with post-translationally modified and unmodified histone H3 or H4 histone tails [61,62,63,64,65,66,67]. Conversely, the atypical PHD of the ZaP domain is a Cys4-His-Cys2-His type zinc finger, which does not bind histones [68,69]. Moreover, a ZaP domain carries a net positive charge that is greater than a PHD domain, suggestive of an interaction with a negatively charged substrate [68]. Indeed, NMR solution and crystal structures that have recently been derived for the BRPF2 and the PHF6 ZaP2 domains indicate that this structure is able to bind double-stranded DNA templates in a sequence-independent manner, however the ability of the ZaP domain to interact with additional nucleic acid structures (e.g., RNA) has so far not yet been investigated [68,69]. Interestingly, a recent high throughput screen identified PHF6 and other ZaP-containing proteins amongst a list of putative mRNA-interactors [70]. Moreover, the PHF6 ZaP1 domain was observed to have nucleolar localization in the absence of the NoLS and a single point mutation of a zinc ion stabilizing residue (C99F) within ZAP1 resulted in the ablation of PHF6 nucleolar localization [59]. In another study, the loss of both NLSs and the NoLS resulted in the nuclear, but not nucleolar, localization of PHF6 [15]. Taken together, these data suggest that additional interactions may be necessary to ensure the recruitment of PHF6 to the nucleoplasm and nucleolus.

### 4.2. PHF6 Interacting Partners

#### 4.2.1 Nucleosome Remodelling and Deacetylation (NuRD) complex

**Figure 2:**
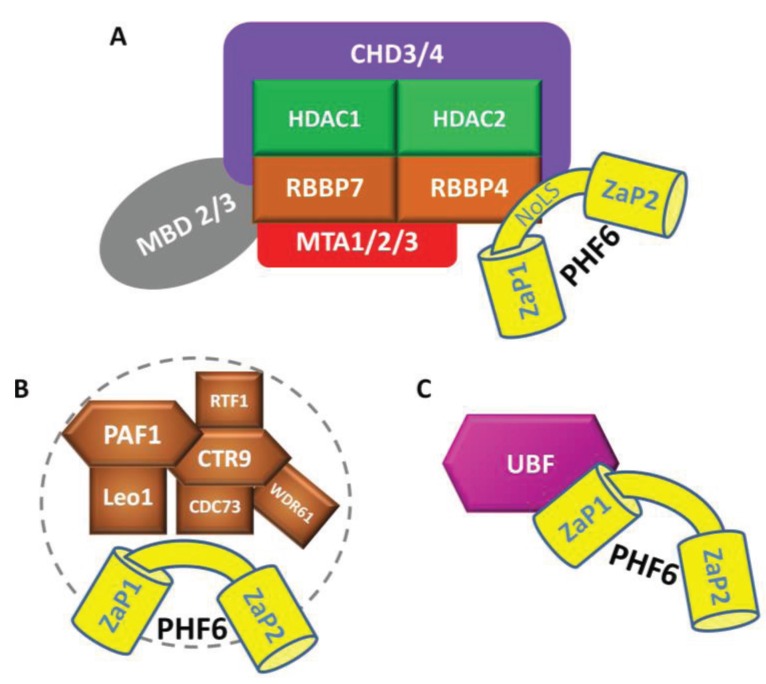
PHF6 interacting proteins. PHF6 associates with (**A**) the NuRD complex via a direct interaction between amino acid residues 162–170 with the β propeller surface of RBBP4; (**B**) the PAF1 complex; and (**C**) UBF, via its ZaP1 domain.

We have previously demonstrated PHF6 to endogenously co-purify with multiple constituents of the NuRD chromatin remodelling complex (CHD3/4, HDAC1, and RBBP4/7) (see Figure 2A), a transcriptional regulator with several gene targets that influence embryogenesis, oncogenesis, neurogenesis, and hematopoiesis [14,71,72,73,74,75,76,77,78]. The NuRD core complex has the ability to catalyze ATP-dependent nucleosome remodelling and histone deacetylation at its gene targets through its CHD and HDAC subunits, respectively [79,80], but the transcriptional outcome of its targets is dependent upon its collective associations with other activators (e.g., P300) and repressors (e.g., LSD1) [81,82]. Several NuRD interactors also possess zinc finger domains with the ability to target the complex to gene targets through DNA sequence-specific interactions (e.g., IKAROS, SALL1, BCL11B), thus the binding affinity of PHF6 to dsDNA may favour the association of NuRD to a distinct subset of its gene targets [83,84,85,86].

**Table 3:** Functional comparison of human proteins expressing PZP motifs and/or ZaP domains.

Protein	Uniprot Accession #	Function	PZP Motif or ZaP Domain	Amino Acid Residues	Reference
PHF6	Q8IWS0	Transcriptional regulation	ZaP (x2)	14–134, 209–332	[14,87]
BRPF1	P55201	MOZ/MORF-dependent H3 acetylation	PZPM	273–450	[88]
BRPF2	O95696	MOZ/MORF-dependent H3 acetylation	PZPM	214–391	[88]
BRPF3	Q9ULD4	MOZ/MORF-dependent H3 acetylation	PZPM	188–389	[88]
G2E3	Q7L622	E3 ubiquitin-protein ligase	ZaP	11–130	[89]
JADE-1	Q6IE81	HBO1-dependent H4 acetylation	PZPM	203–371	[88]
JADE-2	Q9NQC1	HBO1-dependent H4 acetylation	PZPM	199–367	[88]
JADE-3	Q92613	HBO1-dependent H4 acetylation	PZPM	200–368	[88]
JMJD2A (KDM4A)	O75164	H3K9/H3K36 demethylase	PZPM	665–887	[90]
JMJD2B (KDM4B)	O94953	H3K9 demethylase	PZPM	681–909	[90]
JMJD2C (KDM4C)	Q9H3R0	H3K9/H3K36 demethylase	PZPM	642–867	[90]
MLL1 (KMT2A)	Q03164	H3K4 methyltransferase	PZPM	1566–1980	[91]
MLL2 (KMT2D)	O14686	H3K4 methyltransferase	ZaP (x2)	63–220, 5029–5139	[92]
MLL3 (KMT2C)	Q8NEZ4	H3K4 methyltransferase	ZaP (x2)	131–333, 4399–4509	[93]
MLL4 (KMT2B)	Q9UMN6	H3K4 methyltransferase	PZPM	1335–1688	[93]
PHF7	Q9BWX1	Binds chromatin	ZaP	30–147	[94]
PHF11	Q9UIL8	Transcriptional regulation	ZaP	42–162	[95]
PHF14	O94880	Transcriptional regulation	PZPM	319–501	[96]
RAI1	Q7Z5J4	Transcriptional regulation	ZaP	1780–1905	[97]
TCF20	Q9UGU0	Transcriptional regulation	ZaP	1789–1935	[98]

Many NuRD interactors (e.g.,SALL1, BCL11B, FOG-1) interface with the RBBP4 subunit through a conserved amino acid consensus sequence that is shared by PHF6 (aa 152–171) [69,99,100]. Indeed, Liu *et al.* confirmed a direct PHF6-RBBP4 interaction, and subsequently resolved the crystal structure to show that a PHF6 peptide (aa 162–170) peptide occupies a binding pocket located upon the surface of the RBBP4 β propeller [69,101]. Interestingly, the PHF6 NoLS sequence overlaps with the RBBP4 binding domain, suggesting that these two activities may be mutually exclusive. Consistent with this assertion, the PHF6-NuRD/RBBP4 interaction was only found to occur in the nucleoplasm, and not within the nucleolus [14].

#### 4.2.2 RNA Polymerase II Associated Factor 1 (PAF1)

Aside from NuRD, Phf6 interacts with multiple subunits of the Paf1 transcriptional elongation complex (Paf1, Leo1, Cdc73, Ctr9) (see Figure 2B), which is necessary to mediate proper neurogenesis in mice [87]. In this study, *Phf6* and *Paf1* shRNA was electroporated into mouse cerebral cortices, resulting in impaired neuronal migration between E14 and E19. Using gene expression arrays, the authors identified *Neuroglycan C/Chrondroitin Sulfate Proteoglycan 5* (*NGC/CSPG5*) as a commonly regulated gene target, and when the *NGC/CSPG5* transcript was electroporated into cortical tissue alongside *Phf6* shRNA, the neuronal migration phenotype was rescued [87]. These findings are strikingly similar to those of Franzoni *et al.* who also ectopically expressed *Phf6* to rescue neuronal migration defects that accompanied premature expression of miR-128 in the developing neocortices of mice [20]. Interestingly, Paf1 also associates with the RNA Polymerase I machinery that is responsible for transcribing rRNA from rDNA genes, providing a potential means through which nucleolar Phf6 may be able to regulate rDNA gene expression [102].

#### 4.2.3 Additional Interactions

In 2013, two independent studies identified an interaction between PHF6 and UBF (see Figure 2C) [59,87], an rDNA transcriptional activation factor that associates with the RNA Pol I pre-initiation complex [103,104]. In contrast to *NGC/CSPG5*, where PHF6 activates transcription, Wang *et al.* report that PHF6 represses rRNA transcription [59]. Moreover, they also report that a loss of PHF6 results in increased genomic instability at rDNA genes and a cell cycle delay at G2/M. While this was the first indication of a nucleolar function for PHF6, it has also been reported to interact with PRPF8 and SNRNP200, both constituents of the U4/U6-U5 tri-snRNP pre-mRNA splicing ribonucleoprotein complex [14].

### 4.3. PHF6 Is a Putative Phosphoprotein

Aside from these direct studies investigating PHF6 interactions, several phosphoproteomic screens have identified PHF6 peptides, suggesting that it is regulated by phosphorylation [105,106,107,108]. In one study, the PHF6 T358 residue was identified as a target of the ATM DNA damage checkpoint kinase and the loss of PHF6 correlated with the accumulation of phosphorylated γH2AX [10,59,105]. Interestingly, the R342X mutation associated with severe BFLS, and also identified in T-ALL and AML patients would truncate this phosphorylation site. While the functional importance of T358 phosphorylation remains to be determined, one possibility is that it serves as a checkpoint response to negatively regulate rDNA transcription (e.g., via RNA Pol I inhibition) [109].

**Figure 3:**
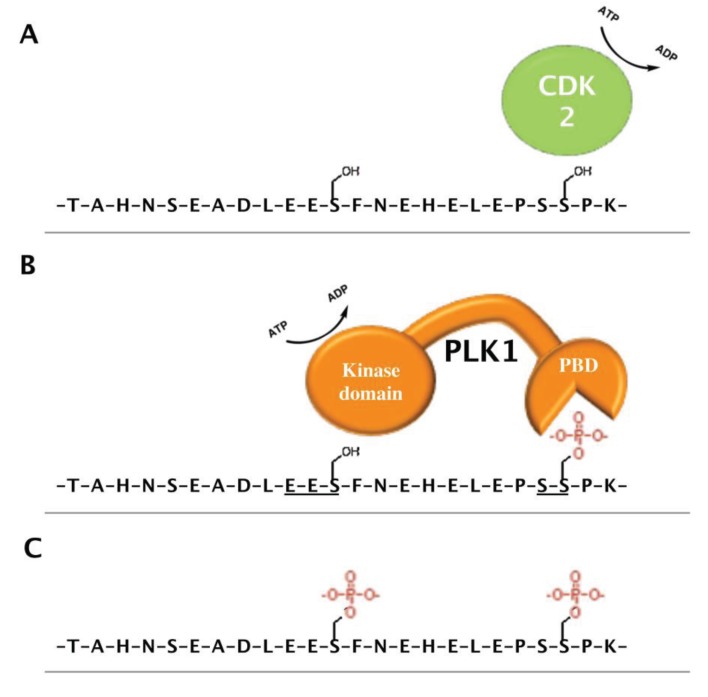
Model for the putative CDK2- and PLK1-dependent phosphorylation of PHF6. Large-scale proteomic studies identified PHF6 Ser-145, -154, and -155 as phosphorylated residues. The phosphorylation of these sites during mitosis or in response to T-cell receptor signalling likely occurs through a mechanism whereby (**A**) CDK2 phosphorylates Ser-155, allowing recognition by the Polo-binding domain (PBD) of PLK1 (**B**), which subsequently phosphorylates Ser-145, which is situated in a PLK1 consensus sequence (143-EESFNE-148), resulting in PHF6 becoming dually phosphorylated at these two sites (**C**).

Two other studies have identified the phosphorylation of three serine residues (S145; S154; and S155) during mitosis and in response to T-cell receptor signalling [106,107]. Interestingly, S145 phosphorylation is only ever observed in combination with phosphorylation at S155. Other high throughput screens indicate that S145 is a Polo-like kinase 1 (PLK1) target; consistent with its situation in a PLK1 consensus sequence; while phosphorylation at S155 provides a binding site for the polo binding domain of PLK1 (PBD); suggesting a mechanism whereby phosphorylation at S155 primes S145 for PLK1-mediated phosphorylation (see Figure 3) [108,110,111]. Indeed PLK1 inhibition reduces S145 phosphorylation with concomitant accumulation of singularly phosphorylated S155 peptide [108,112]. Interestingly, another screen identified S155 as a candidate substrate for CDK2 phosphorylation, and CDK2 inhibition interferes with the ability of PHF6 to localize to the nucleolus [113,114]. While the exact mechanism of this serine phosphorylation network within PHF6 requires more direct validation; it should be noted that PLK1 and CDK2 are highly active during S and G_2_ phase when nucleolar size and the rate of ribosome biogenesis is higher relative to G_1_ [115,116]. Moreover, these phosphorylation sites are directly adjacent to the NoLS. Thus, we speculate that these phosphorylation events may promote shuttling of PHF6 between the nucleoplasm and nucleolus in response to external needs, such as compensation for the increased rates of rRNA synthesis that precede cytokinesis or following T-cell receptor activation during lymphogenesis.

## 5. Perspectives and Future Directions

### 5.1. Predicting Clinical Outcomes for Patients Expressing PHF6 Loss-of-Function

The PHF6 protein is important for both faithful development and tumour suppression, yet only 6.5% of germline *PHF6* mutations (see Table 1) are associated with cancer [29,31]. To explain why not all BFLS patients develop cancer, one consideration is that BFLS patients primarily have missense mutations that are likely functionally hypomorphic, whereas somatic *PHF6* mutations causing cancer are frameshift, nonsense, or deletions that are in all likelihood functionally null. In addition, BFLS patients would still require a second mutation (e.g., *TLX1*) within hematopoietic cells. Since female Coffin–Siris patients have a similar type of mutation to those individuals with T-ALL, they may be at greater risk for developing cancer. However, female patients with *de novo* germline deletions of *PHF6* all have skewed X-chromosome inactivation in the blood, which may explain why hematopoietic cancers have so far not been observed. 

### 5.2. Developmental Role of PHF6

**Figure 4:**
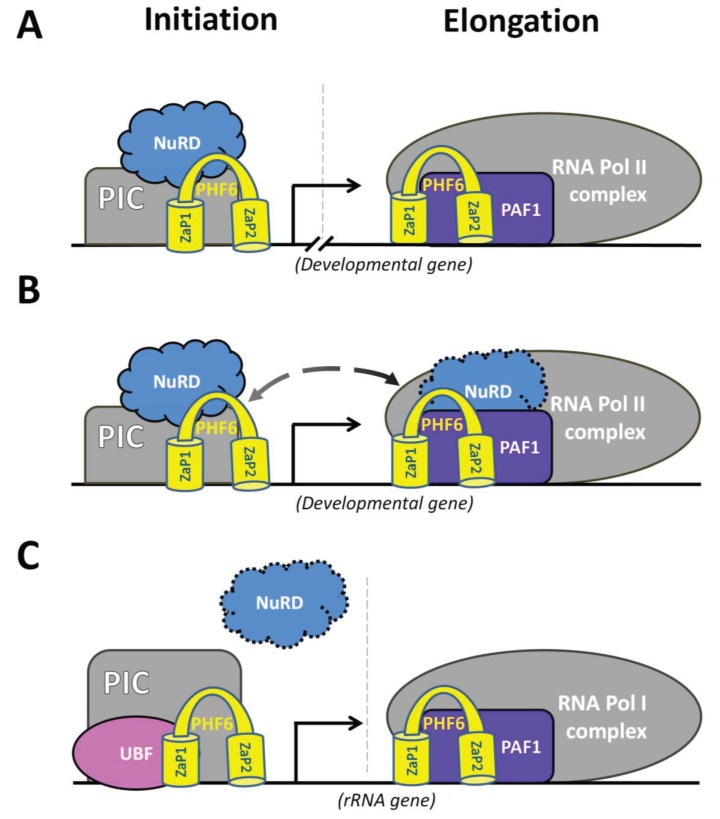
Model for PHF6-dependent transcriptional regulation at developmental or rDNA gene targets. (**A**) At developmental genes, PHF6 recruits NuRD to promoters to either activate or repress transcription. Similarly, PHF6 can regulate transcriptional elongation at developmental genes through its interaction with the PAF1 complex. (**B**) Another possible mechanism is that PHF6 interactions with NuRD and PAF1 occur at the same gene whereby PHF6 promotes the formation of a NuRD-PAF1 supercomplex to allow for productive elongation. The existence of a PHF6-mediated supercomplex would be analogous to an IKAROS-driven complex previously described [119]. (**C**) At rRNA genes, PHF6 interacts with UBF to mediate the initiation of rRNA transcription (left). Promoter regulation may also involve NuRD, although this remains to be shown. Similarly, PHF6-PAF1 may mediate rRNA transcriptional elongation, although experimental validation is still required.

The PHF6 interactors identified to date suggest that PHF6 is a multifaceted regulator of transcription working with the NuRD chromatin remodelling complex (or UBF) to repress or activate transcriptional initiation, and with the PAF1 complex in controlling transcriptional elongation (Figure 4A). While these events may appear as disparate aspects of transcriptional control, they could be intimately linked. Indeed, many studies of IKAROS, a chromatin adaptor protein with DNA-binding zinc finger motifs, show that it can interact with both NuRD and the CDK9 catalytic subunit of positive-transcription elongation factor b (P-TEFb) [117,118,119]. Recent work has shown that IKAROS can recruit NuRD to specific target genes but it also interacts in a larger complex containing both NuRD and P-TEFb, in which IKAROS permits the transfer of protein phosphatase 1α (PP1α) to CDK9, which subsequently phosphorylates Ser-2 residues in the RNA pol II C-terminal domain (CTD), releasing RNA Pol II from promoter-proximal pausing to initiate elongation [119]. Thus, it is possible that PHF6 may interact within a NuRD-PAF1 supercomplex to promote productive elongation (see Figure 4B), although the existence of such a complex has yet to be defined.

In conducting future investigations to elucidate the transcriptional roles of such PHF6-containing complexes, it will be important to identify all PHF6 target genes through tissue-specific ChIP-Seq as a starting point to assess which PHF6 targets are PAF1 regulated, which ones require the recruitment of NuRD, and which ones might utilize both complexes. Already, ChIP-Seq experiments for the NuRD constituents CHD4 and MBD3 indicate an enrichment of binding sites in gene bodies and well-defined peaks at transcriptional start site (TSS) regions [78,120]. Similarly, a recent PHF6 ChIP-Seq dataset generated from Jurkat cells presents binding sites that are predominantly located throughout gene bodies and within the proximal promoter [58]. Nonetheless, dissecting the different mechanisms of transcriptional regulation through the identification of target genes will allow for the better definition of relevant developmental pathways (e.g., neurogenesis, hematopoiesis) that become compromised in patients with BFLS, T-ALL, and/or AML. In this regard, the recent findings of a network whereby neurogenesis depends upon the timely expression of *PHF6* to positively regulate *NGC/CSPG5* (via PAF1) to ensure proper cortical neuron migration, and miR-128 to negatively regulate *PHF6*, which allows for subsequent neuronal maturation, represent the first clear model of a developmental role for *PHF6* [20,87]. Moreover, PHF6 binding sites from the Jurkat ChIP-Seq dataset are found within genes responsible for cell cycle regulation, cell morphology mechanisms, and cell signalling pathways mediating axonal guidance [58]. Interestingly, PHF6 binds the *NOTCH1* and *RUNX1* promoters, genes that have mutations that are co-expressed with *PHF6* mutations in T-ALL and AML, respectively [8,48]. Furthermore, the fact that NOTCH1 was itself found to bind the *PHF6* promoter in a T-ALL cell line suggests that hematopoietically expressed PHF6 may participate within a feedback regulation network [22]. Therefore, carrying out these types of multidisciplinary approaches in primary tissues will be crucial to more fully understanding the functional roles of PHF6.

### 5.3 Nucleolar Role of PHF6

In addition to developmental gene targets, PHF6 may utilize similar complexes to control rDNA transcription, as outlined in Figure 4. In this regard, both NuRD and PAF1 have been demonstrated to contribute to the transcriptional competency of rDNA genes [102,121,122]. Moreover, control of rRNA levels may also involve nucleolar localization of PHF6 since the NuRD interaction motif overlaps with the NoLS and is directly adjacent to the Ser 154/155, which were proposed to be phosphorylated by CDK2 and whose inhibition prevents PHF6 from localizing to the nucleolus [101,113,114]. While it is an attractive hypothesis, further experiments are still required to delineate the importance of PHF6 to the regulation of ribosome biogenesis. Still, the transcription of rRNA accounts for as much as half of all cellular transcription, with ribosome biogenesis rates correlating positively to nucleolar size [59,123].

In many cancers, it is the hyperactivation of ribosome biogenesis that contributes to the pathology, with large nucleoli correlating to poorer clinical outcomes [124]. Given that PHF6 is reported as a negative regulator of rDNA transcription, PHF6 loss-of-function is consistent with increased ribosome biogenesis and contributing to clonal growth in T-ALL. Indeed several T-ALL mutations have been identified in genes encoding ribosomal proteins (e.g.; *RPL5, RPL10, RPL11, RPL22*), whereas others (e.g., *NOTCH1*, *PTEN, FBXW7*) target aspects of the cap-dependent mRNA translation machinery, as has been the subject of recent reviews [125,126]. For some of these genes, a mechanism supporting translational hyperactivation remains unclear, with De Keersmaecker and colleagues arguing for a model where it is the reduction of translational fidelity that contributes to tumorigenesis [125,126]. It may also be the case that defective ribosomes, or their individual subunits, have altered extraribosomal functions, allowing tumour cells to overcome cell cycle checkpoints or interfere with tissue-specific developmental pathways [126,127]. In this regard, interactions between PHF6 and ribosomal proteins have been identified and as such, may also impinge upon ribosomal and extraribosomal functions [14].

In neurons, large nucleoli facilitate the sufficient supply of ribosomes to growing neurites to accommodate local demands for translation [128]. Indeed many developmental intellectual disability diseases (e.g., Cockayne syndrome, Rett syndrome) and acquired neurodegenerative disorders (e.g., Alzheimer’s disease, Parkinson’s disease) are associated with reduced levels of ribosome biogenesis [129,130]. Although hyperactive ribosome biogenesis would appear paradoxical with BFLS, a potential explanation may exist by considering the loss of MeCP2, which is responsible for Rett syndrome [131]. In this example, MeCP2 binds methylated CpG DNA, a mark for rDNA silencing [132], and contributes to heterochromatin formation [133]; yet, rather than hyperactivating rRNA synthesis, the loss of MeCP2 correlates with reduced nucleolar size [134].

## 6. Conclusion

Since the discovery of PHF6 as the cause of BFLS in 2002, the precise function of the protein has remained elusive. Nonetheless, it is becoming clear that PHF6 is a highly dynamic chromatin adaptor protein, containing two ZaP domains that facilitate its interactions with nucleic acids and a growing number of interaction partners (NuRD, PAF1, UBF) to regulate transcription. Moreover, several potential phosphorylation sites, its interaction with rDNA, and its ability to shuttle between the nucleolus and nucleoplasm suggest that it may be a key regulator of ribosome biogenesis. The generation of transgenic mouse models with BFLS-like, T-ALL-like, or AML-like *PHF6* mutations, will certainly augment the dissection of the molecular mechanisms driving BFLS and leukemia. In making use of such models, further characterization of the functional roles of PHF6 may thus facilitate the development of novel therapeutics for these distinct disorders. 
